# Improvements in Diet and Physical Activity–Related Psychosocial Factors Among African Americans Using a Mobile Health Lifestyle Intervention to Promote Cardiovascular Health: The FAITH! (Fostering African American Improvement in Total Health) App Pilot Study

**DOI:** 10.2196/28024

**Published:** 2021-11-12

**Authors:** Jissy Cyriac, Sarah Jenkins, Christi A Patten, Sharonne N Hayes, Clarence Jones, Lisa A Cooper, LaPrincess C Brewer

**Affiliations:** 1 Department of Internal Medicine Mayo Clinic School of Graduate Medical Education Rochester, MN United States; 2 Division of Clinical Trials and Biostatistics Department of Quantitative Health Sciences Mayo Clinic Rochester, MN United States; 3 Department of Psychiatry and Psychology Mayo Clinic College of Medicine Rochester, MN United States; 4 Department of Cardiovascular Medicine Mayo Clinic College of Medicine Rochester, MN United States; 5 Hue-Man Partnership Minneapolis, MN United States; 6 Department of Health, Behavior and Society Johns Hopkins Bloomberg School of Public Health Baltimore, MD United States; 7 Center for Health Equity and Community Engagement Research Mayo Clinic Rochester, MN United States

**Keywords:** African Americans, cardiovascular health disparities, mHealth lifestyle intervention, diet, physical activity, mobile phone

## Abstract

**Background:**

African Americans continue to have suboptimal cardiovascular health (CVH) related to diet and physical activity (PA) behaviors compared with White people. Mobile health (mHealth) interventions are innovative platforms to improve diet and PA and have the potential to mitigate these disparities. However, these are understudied among African Americans.

**Objective:**

This study aims to examine whether an mHealth lifestyle intervention is associated with improved diet and PA-related psychosocial factors in African Americans and whether these changes correlate with diet and PA behavioral change.

**Methods:**

This study is a retrospective analysis evaluating changes in diet and PA-related self-regulation, social support, perceived barriers, and CVH behaviors (daily fruit and vegetable intake and moderate-intensity PA [MPA] per week) in 45 African American adults (mean age 48.7 years, SD 12.9 years; 33/45, 73% women) enrolled in the FAITH! (Fostering African American Improvement in Total Health) app pilot study. The intervention is a 10-week, behavioral theory–informed, community-based mHealth lifestyle intervention delivered through a mobile app platform. Participants engaged with 3 core FAITH! app features: multimedia education modules focused on CVH with self-assessments of CVH knowledge, self-monitoring of daily fruit and vegetable intake and PA, and a sharing board for social networking. Changes in self-reported diet and PA-related self-regulation, social support, perceived barriers, and CVH behaviors were assessed by electronic surveys collected at baseline and 28 weeks postintervention. Changes in diet and PA-related psychosocial factors from pre- to postintervention were assessed using paired 2-tailed *t* tests. The association of changes in diet and PA-related psychosocial variables with daily fruit and vegetable intake and MPA per week was assessed using Spearman correlation. Associations between baseline and 28-week postintervention changes in diet and PA-related psychosocial measures and CVH behaviors with covariates were assessed by multivariable linear regression.

**Results:**

Participants reported improvements in 2 subscales of diet self-regulation (decrease fat and calorie intake, *P*=.01 and nutrition tracking, *P*<.001), one subscale of social support for healthy diet (friend discouragement, *P=*.001), perceived barriers to healthy diet (*P*<.001), and daily fruit and vegetable intake (*P*<.001). Improvements in diet self-regulation (increase fruit, vegetable, and grain intake, and nutrition tracking) and social support for healthy diet (friend encouragement) had moderate positive correlations with daily fruit and vegetable intake (*r*=0.46, *r*=0.34, and *r*=0.43, respectively). A moderate negative correlation was observed between perceived barriers to healthy diet and daily fruit and vegetable intake (*r*=−0.25). Participants reported increases in PA self-regulation (*P*<.001). Increase in social support subscales for PA (family and friend participation) had a moderate positive correlation with MPA per week (*r*=0.51 and *r*=0.61, respectively).

**Conclusions:**

Our findings highlight key diet and PA-related psychosocial factors to target in future mHealth lifestyle interventions aimed at promoting CVH in African Americans.

## Introduction

### Background

African Americans have the highest rates of coronary heart disease and stroke-related deaths compared with White people and other racial and ethnic minority groups in the United States [1]. Cardiovascular risk factors such as suboptimal diet and physical activity (PA) have been identified as significant contributors to the disproportionate cardiovascular disease (CVD) burden among African Americans [1]. African Americans have an extremely low prevalence of individuals achieving ideal levels on 5 or more cardiovascular health (CVH) metrics, as outlined by the American Heart Association Life’s Simple 7 (LS7) [1-3].

Suboptimal CVH in African Americans is rooted in structural racism, which has systematically limited their access to quality health care, employment opportunities, education, and safe neighborhood environments. The resulting scarcity of resources has led to elevated stress levels, food insecurity, and poor access to recreational spaces, all of which affect CVH in African Americans [4-7]. These structural inequities can further manifest as negative psychosocial factors that can influence health behaviors and CVH outcomes in African Americans [8-11]. In African Americans, increased self-reported stress and depressive symptoms have been associated with greater calorie consumption and lower levels of PA, respectively [10]. There is evidence to suggest that psychosocial factors affecting healthy diet and PA, such as cost and lack of time, are negatively associated with diet quality and regular PA, respectively [12-14]. Data on diet-related psychosocial factors indicate that self-regulatory behaviors, such as mindful food preparation, have been associated with healthier food acquisition and decreased purchase of preprepared foods among African American adults [15,16]. In addition, culturally tailored interventions integrating social support and addressing barriers have been identified as facilitators of PA in African American women [14,17]. African American women have also expressed a preference for interventions that promote self-regulatory behaviors (self-monitoring of PA) and incorporate strategies to overcome barriers to improve their PA patterns [17]. These findings provide compelling evidence that targeting diet and PA-related self-regulation, social support, and perceived barriers in mobile health (mHealth) lifestyle interventions for African Americans may facilitate improvements in diet and PA behaviors.

Mobile app–based health interventions have the potential to improve diet and PA patterns among African Americans. Research from the Pew Research Center has shown that African Americans are less likely than White people to have traditional broadband access at home but are equally likely to own cell phones and smartphones. They are also more likely to use smartphones to access the internet, web-based social networking sites, and health information [18-20]. Thus, the use of smartphones for health promotion has the potential to mitigate health disparities, and makes progress toward achieving health equity through technology-based interventions [21].

In addition, there is emerging evidence that interventions using mHealth apps may promote diet and PA behavior change [22-24] and may be effective interventional tools for underserved African American communities [21,25]. However, mHealth interventions have been significantly understudied in African American populations. James et al [26] found that <10% of identified mHealth studies within their systematic review included African American participants, and only 14% of those mHealth studies entirely comprised all African American participants. This is despite African Americans having high smartphone ownership, mobile technology use (including mobile apps), and eHealth literacy (EHL) [20,27-29]. EHL is defined as an individual’s ability to search for and understand health information on the internet using computers and mobile devices [30]. Given high EHL and smartphone ownership in African Americans, mHealth lifestyle interventions may be particularly effective in promoting healthy diet and PA behaviors.

Current mHealth interventions to promote healthy diet and PA in African Americans suggest that they can be effective as stand-alone interventions [31-35] or adjuncts to in-person interventions [36-39]. Allicock et al [31] demonstrated that a stand-alone, mobile app–based intervention to encourage healthy diet and PA behaviors among African American survivors of breast cancer led to a significant reduction in sedentary time and fast food intake. Another culturally tailored, internet-based intervention for PA promotion as an adjunct to in-person PA sessions resulted in a significant reduction in sedentary behaviors among African American women [38]. Furthermore, mHealth interventions have demonstrated success in increasing diet and PA self-monitoring [39], promoting healthy diet and regular PA [33,35] and facilitating weight loss in African Americans [32,34,36,37]. Although mHealth lifestyle interventions have demonstrated improvements in diet and PA outcomes, few have discussed the interplay between psychosocial factors such as underlying diet and PA-related self-regulation, social support, perceived barriers, and their associations with health behaviors [33,34,38,40].

### Goal of This Study

We seek to provide insight into this current gap in the literature by exploring changes in diet and PA-related self-regulation, social support, perceived barriers, and their associations with diet and PA patterns among African Americans using our culturally tailored, community-based mHealth lifestyle intervention. The FAITH! (Fostering African American Improvement in Total Health) app pilot study tested a novel mHealth intervention aiming to improve CVH in African Americans through local African American church congregations [41]. Through the use of the FAITH! app, participants had statistically significant improvements in diet, PA, blood pressure, and overall composite LS7 score [35].

The primary aim of this study is to examine changes in diet and PA-related self-regulation, social support, and perceived barriers among participants in the FAITH! app pilot study. We also aim to assess whether any of the observed changes in these measures correlate with CVH behaviors (daily fruit and vegetable intake and moderate-intensity PA [MPA] per week). We hypothesize that participants would demonstrate improvements in diet and PA-related self-regulation, social support, perceived barriers, and CVH behaviors using the FAITH! app intervention.

## Methods

### Study Design

This study is a retrospective analysis examining changes in diet and PA-related psychosocial factors in the FAITH! app pilot study. Details of the intervention design, recruitment, implementation, and outcomes have been previously published [35,41,42]. Briefly, the pilot study was conducted using a single group, pretest-posttest intervention framework to assess CVH knowledge, behaviors, and biological factors among African Americans following the use of a mobile app–based intervention. The cohort was recruited from 5 African American churches within the Rochester and Minneapolis-St. Paul, Minnesota, metropolitan areas. The pilot study was registered with the Clinical Trials Registry (ClinicalTrials.gov NCT03084822) and approved by the Mayo Clinic institutional review board. Participants provided written informed consent before enrollment in the study.

### Data Collection

Baseline data were collected in July 2016, and follow-up data at 28 weeks were collected postintervention (April 2017). Electronic surveys were emailed directly to participants to assess their sociodemographic data, EHL, diet and PA-related self-regulation, social support, and perceived barriers, along with CVH behaviors (daily fruit and vegetable intake and MPA per week) at baseline and 28 weeks post intervention. Postintervention evaluation occurred at 28 weeks in an effort to align with the AHA LS7, the primary outcome of the parent study [35]. The LS7 comprises both biological and behavioral factors, and its evaluation was completed at the 28-week postintervention time point to assess for sustained changes in these variables. Thus, the present analysis examines changes from baseline to 28 weeks postintervention to concurrently assess for sustained changes in diet and PA-related self-regulation, social support, perceived barriers, and CVH behaviors. Outcome evaluation was designed based on psychosocial measures previously described in the literature, noted trends in a previous in-person iteration of the FAITH! program, and the theoretical foundation of the FAITH! app intervention [42-44]. Participants received up to US $150 in gift cards for their completion of survey assessments.

### Theoretical Framework: Intervention and Psychosocial Measures

**Figure 1:**
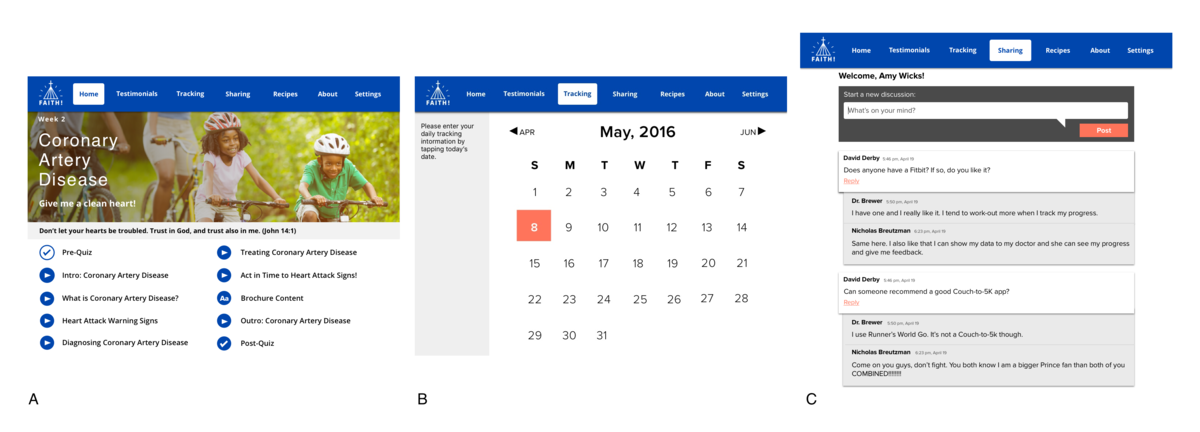
FAITH! (Fostering African American Improvement in Total Health) app core features: (A) cardiovascular health education modules and self-assessments; (B) diet and physical activity self-monitoring; (C) sharing board.

The 10-week intervention was delivered through a mobile app (FAITH! app; Figure 1) and consisted of 3 core features: (1) a multimedia education module series on CVH with pre- and postmodule self-assessments of CVH knowledge, (2) self-monitoring of daily fruit and vegetable intake and PA, and (3) a sharing board for networking with other participants. At study enrollment, participants were provided iPads with the FAITH! app installed and Fitbits (Charge 2, 2016 version, Fitbit Inc) for PA and step tracking. Within the FAITH! app, participants were expected to complete one educational module per week and complete the associated pre- and postmodule self-assessments, with completion of all educational modules by the end of the 10-week intervention. Participants entered daily entries for fruit and vegetable intake and PA during the course of 10 weeks. In addition, participants were encouraged to post on the sharing board for social networking with other study participants; no limits were imposed on the number of posts an individual participant could submit. Participants were expected to engage in all 3 core app features described above for the 10-week intervention delivery phase, and their engagement patterns with these features were closely monitored via Google Analytics [45]. Participants continued to have access to the app in the postintervention phase, but this was not monitored. Further details of the FAITH! app design and key features have been previously published [42].

The FAITH! app was developed using a community-based participatory approach grounded in behavioral theories (social cognitive theory, health belief model, and community mobilization model) [42]. Social cognitive theory posits that individual health behavior is dependent on their reciprocal interaction with observational learning and reinforcements. We applied this theory within the FAITH! app via the sharing board, where participants could see others from their own communities modeling positive health behaviors (eg, diet and PA). This in turn could motivate their adoption of these behaviors [42]. In addition, the sharing board allowed individuals to receive positive reinforcement from others in the study to maintain healthy diet and PA behaviors. Correspondingly, social support was assessed to gauge the degree of positive reinforcement and observational role modeling experienced by participants through the use of the intervention. Central to the health belief model is that interventions are more effective in changing health behaviors if they influence an individual’s perception of susceptibility to illness or disease, severity of illness, potential positive benefits of healthy action, barriers to such action, and exposure to factors that prompt action (cues to action) [46]. Furthermore, a focus on perceived barriers and perceived benefits has been demonstrated to be the strongest predictor of behavior change [47]. On the basis of the principles of the health belief model, the CVH education modules were designed to convey the high CVD risk of African Americans and the severity of the consequences of CVD (heart attacks, heart failure, and strokes). In addition, the modules emphasized greater benefits than barriers to maintaining a healthy diet and regular PA and thus aimed to increase the likelihood that participants would take action to engage in these health behaviors. Participants’ prompts to action and self-directed behavior were further reinforced by encouraged use of the diet and PA self-monitoring app features. Accordingly, changes in diet and PA self-regulation and perceived barriers to healthy diet and PA were assessed to determine how effectively the intervention addressed these components of the health belief model [42]. Community mobilization was leveraged with the involvement of community partners from participating African American churches in all stages of app design using a community-based participatory research approach to ensure that the app incorporated African American religious and spiritual beliefs, the social connectedness aspects of the African American church, and cultural traditions of the African American community [42,48]. African American churches have been the cornerstone of academic-community partnerships seeking to enact social justice through research to improve health and access to quality care among African Americans [49].

### Measurement of Psychosocial Factors

#### Diet-Related Measures

Diet self-regulation was assessed using 14 items from the Health Beliefs Survey developed by Anderson et al [50], where each item was measured using a 5-point Likert scale (1=never to 5=always). Three subscales of diet self-regulation were assessed, including strategies to (1) increase fruit, vegetable, and grain intake (3 survey items; Cronbach α=.67), (2) decrease fat and calorie intake (6 survey items; Cronbach α=.78), and (3) plan and track nutrition (5 survey items; Cronbach α=.80). Responses for items in each category were averaged to calculate each score (range 1-5). Higher scores indicated greater dietary self-regulation. Social support for healthy diet was assessed using a survey instrument developed by Sallis et al [51], which consists of 20 items rated on a 5-point Likert scale (1=never to 5=very often) that assessed subscales of encouragement and discouragement from family and friends for healthy diet (4 subscales: family encouragement [5 items; Cronbach α=.81], friend encouragement [5 items; Cronbach α=.81], family discouragement [5 items; Cronbach α=.56], and friend discouragement [5 items; Cronbach α=.74]). Higher scores implied greater encouragement or discouragement, respectively. Perceived barriers to healthy diet were assessed using 15 items adapted from the Lose It Forever study questionnaire by Welsh et al [52]. The survey asked participants to evaluate the availability of healthy foods, the ability to control cravings for unhealthy foods, prepare healthy meals, and level of difficulty in the daily environment to choose healthy foods. Participants responded to items on a 5-point Likert scale (1=not at all true to me to 5=very true for me). Responses for each item were averaged to calculate a final score (range 1-5), with higher scores implying greater barriers (Cronbach α=.88).

#### PA-Related Measures

PA self-regulation was measured using the Self-Regulation Scale from the Health Beliefs Survey, which includes 10 items, using a 5-point Likert scale (1=never to 5=always) [33,50,53]. The scale assesses strategies to increase PA and the efforts used to track step counts over the past month. Scores across all items were averaged to calculate a final score (range 1-5), with higher scores indicating higher PA self-regulation (Cronbach α=.84). Social support for PA was measured using a survey instrument developed by Sallis et al [51] and comprised 23 items rated on a 5-point Likert scale (1=never to 5=very often) that assessed subscales of: family participation [10 items; Cronbach α=.92], family rewards or punishment [3 items; Cronbach α=.48], and friend participation [10 items; Cronbach α=.93]. Higher scores implied greater participation or reward or punishment, respectively. An adaptation of the Exercise Barriers Scale was used to assess barriers to exercise [54,55]. Participants rated a list of 20 items on a 4-point Likert scale (1=strongly agree to 4=strongly disagree) on how much they presented a barrier to exercise. Items included time for PA, convenience to complete PA, competing responsibilities, and concerns about hairstyle. The response to each item was reversed so that more agreement indicated more barriers. The items were averaged to calculate the final score (range 1-4), with higher scores implying greater barriers (Cronbach α=.91).

### CVH Behaviors

Self-reported daily fruit and vegetable intake was assessed using 2 items adapted from previously developed instruments assessing fruit and vegetable intake [56-58]. Respondents reported daily servings of fruit and vegetables consumed. This instrument has been previously validated in a similar population of African Americans [59]. PA was assessed as self-reported total minutes of MPA per week using the short form of the International Physical Activity Questionnaire) survey. The International Physical Activity Questionnaire has also been validated among African Americans [60,61]. MPA per week was used as it is a part of the standardized LS7 components and associated metrics [2,35].

### Covariates

Key covariates for assessment of associations with psychosocial factors as well as diet and PA behaviors included sociodemographic data (age, sex, income, education level, marital status, and employment status), EHL, and level of app engagement. Participant EHL was measured using the eHealth Literacy Scale, which consists of 8 items on a 5-point Likert scale (1=strongly disagree to 5=strongly agree; Cronbach α=.89) assessing self-reported skills in using eHealth information [30]. High versus low EHL was dichotomized at a score of 26, as previously used by Richtering et al [62]. The level of app engagement was categorized as high versus low. Participants with high app engagement met at least two of the following three criteria: (1) >70% completion of self-assessments within CVH education modules, (2) at least 7 entries into the diet and PA self-monitoring feature, and (3) at least one post on the sharing board. Participants who did not meet these criteria were categorized as having low app engagement. These parameters were determined by real-time monitoring of the participants’ use patterns of key features within the FAITH! app throughout the intervention phase (via Google Analytics). As a pilot study of the newly created FAITH! app among a fairly understudied population, a priori engagement patterns were not available.

### Statistical Analyses

Participant data were summarized with frequencies and percentages, means and SDs, or medians and IQRs, as appropriate. Changes in the psychosocial measures from baseline to 28 weeks postintervention (∆) were assessed with paired 2-tailed *t* tests, with the exception of MPA per week, which was assessed with the signed-rank test. Effect sizes (Cohen *d*) were calculated as the average difference (28 weeks postintervention minus the baseline) divided by the SD of the difference, with the exception of MPA per week, for which the effect size was calculated as the difference in medians divided by the IQR. Effect sizes of <0.5 were categorized as *small*, 0.5 to <0.8 was categorized as *medium*, and ≥0.8 was categorized as *high*. Associations between baseline and 28-week postintervention changes in psychosocial measures and CVH behaviors with participant covariates (sex, employment status, EHL, and app engagement) were assessed using multivariable linear regression (or quantile regression at the median for MPA per week), including all covariates together. Given the small sample size, a focused set of covariates was included in the multivariable analysis. Associations between changes in diet and PA-related psychosocial measures with changes in daily fruit and vegetable intake and MPA per week were quantified using Spearman correlations (*r*). Correlations >0.2 were considered moderately associated, and correlations of >0.7 were considered highly associated. All analyses were performed using the SAS (version 9.4; SAS Institute, Inc). All statistical tests were 2-tailed, and statistical significance was defined as *P*≤.01, and 99% CIs were reported along with the mean differences.

## Results

### Participant Demographics

The analytic sample included 45 African American participants (mean age 48.7 years, SD 12.9 years; 33/45, 73% women) who completed the 10-week mHealth lifestyle intervention and surveys at baseline and 28-week postintervention (Table 1).

### Changes in Diet and PA-Related Psychosocial Measures and CVH Behaviors

#### Overview

Table 2 summarizes changes in the sample for all measured diet and PA-related psychosocial measures along with CVH behaviors from baseline to 28 weeks postintervention.

**Table 1:** Baseline participant characteristics (N=45).

Characteristics		Values
Age (years), mean (SD; range)		48.7 (12.9; 26.0-72.0)
**Sex** **, n (%)**		
	Women	33 (73)
	Men	12 (27)
**Annual household income (n=40; US $)** **, n (%)**		
	<35,000	14 (35)
	≥35,000	26 (65)
**Employment status, n (%)**		
	Employed at least part time	34 (76)
	Unemployed	11 (24)
**Marital status, n (%)**		
	Unmarried	22 (49)
	Married	23 (51)
**Education level, n (%)**		
	No degree	15 (33)
	Technical, associate’s, college, or advanced degree	30 (67)
**eHealth literacy score (n=40)**		
	Value, mean (SD; range)	30.5 (4.5; 21.0-40.0)
	Low (<26), n (%)	6 (15)
	High (≥26), n (%)	34 (85)
**App engagement, n (%)**		
	Low	20 (44)
	High	25 (56)

**Table 2:** Changes in diet and physical activity (PA)-related psychosocial measures and cardiovascular health behaviors, baseline to 28 weeks postintervention (N=45).

Characteristics				Baseline, mean (SD)^a^		28 weeks postintervention, mean (SD)^a^		Difference in score^a,b^, mean (SD; 99% CI)		Effect size^c^ (Cohen *d*)		*P* value
**Diet-related psychosocial measures**												
	Diet self-regulation											
		Increase fruit, vegetable, and grain intake	3.3 (0.7)		3.5 (0.7)		0.2 (0.8; −0.1 to 0.6)		0.32		.04	
		Decrease fat and calorie intake	3.0 (0.7)		3.3 (0.7)		0.3 (0.8; 0.0 to 0.6)		0.41		.01	
		Nutrition tracking	2.1 (0.8)		2.7 (0.8)		0.6 (0.9; 0.2 to 1.0)		0.61		<.001	
	Social support for healthy diet											
		Family encouragement	13.4 (4.8)		12.9 (5.2)		−0.5 (3.6; −2.0 to 1.0)		−0.14		.36	
		Family discouragement	12.0 (3.8)		11.5 (4.7)		−0.5 (3.9; −2.1 to 1.1)		−0.14		.38	
		Friend encouragement	11.8 (4.6)		10.9 (4.7)		−1.0 (5.0; −3.2 to 1.3)		−0.19		.25	
		Friend discouragement	11.2 (4.6)		9.0 (3.2)		−2.1 (3.6; −3.7 to −0.5)		−0.60		.001	
	Perceived barriers to healthy diet			2.5 (0.7)		2.2 (0.6)		−0.4 (0.5; −0.6 to −0.2)		−0.76		<.001
**Diet behavior**												
	Daily fruit and vegetable intake (servings per day)			3.4 (1.4)		4.5 (1.8)		1.2 (1.9; 0.4 to 1.9)		0.62		<.001
**PA-related psychosocial measures**												
	PA self-regulation			2.3 (0.6)		2.7 (0.7)		0.4 (0.7; 0.2 to 0.7)		0.65		<.001
	Social support for PA											
		Family participation	19.7 (8.9)		20.1 (8.8)		0.3 (8.6; −3.2 to 3.8)		0.04		.80	
		Family rewards or punishment	3.7 (1.5)		4.0 (1.6)		0.4 (1.5; −0.2 to 1.0)		0.25		.10	
		Friend participation	18.5 (10.2)		21.9 (9.4)		3.4 (8.4; −0.8 to 7.6)		0.40		.03	
	Perceived barriers to PA			1.7 (0.4)		1.7 (0.5)		0.0 (0.5; −0.2 to 0.2)		−0.01		.93
**PA behavior**												
	MPA^d^ per week (minutes per week), median (IQR; 99% CI)^e^			35.0 (0.0 to 110.0)		75.0 (25.0 to 187.5)		30.0 (−12.5 to 122.5; −52.5 to 92.5)		0.22		.04

^a^Mean (SD) shown, unless otherwise specified.

^b^Difference in score calculated before rounding as change in score from baseline to postintervention.

^c^Effect size calculated before rounding as the mean difference divided by the SD of the difference, unless otherwise specified.

^d^MPA: moderate-intensity physical activity.

^e^99% CI for median difference estimated with quantile regression; effect size calculated as median difference divided by IQR; *P* value from signed-rank test.

#### Diet-Related Psychosocial Measures and Daily Fruit and Vegetable Intake

Participants reported statistically significant improvements in 2 subscales of diet self-regulation (decrease fat and calorie intake: ∆ +0.3; *P*=.01; Cohen *d*=0.41 and nutrition tracking: ∆ +0.6; *P*<.001; Cohen *d*=0.61), one subscale of social support (friend discouragement: ∆ −2.1; *P*=.001; Cohen *d*=−0.60), and perceived barriers to healthy diet (∆ −0.4; *P*<.001; Cohen *d*=−0.76) from baseline to 28 weeks postintervention. The sample also showed statistically significant improvements in reported daily fruit and vegetable intake (∆ +1.2; *P*<.001; Cohen *d*=0.62).

#### PA-Related Psychosocial Measures and MPA Per Week

Participants reported statistically significant improvements in PA self-regulation (∆ +0.4; *P*<.001; Cohen *d*=0.65) from baseline to 28 weeks postintervention. Participants reported a slight improvement in MPA per week (∆ +30 minutes; *P*=.04; Cohen *d*=0.22); however, this did not reach statistical significance.

### Correlation of Psychosocial Measures With CVH Behaviors

Tables 3 and 4 summarize the correlation of the measured changes in diet and PA-related self-regulation, social support, and perceived barriers to CVH behaviors. Improvements in subscales of diet self-regulation (increase fruit, vegetable, and grain intake and nutrition tracking) had a moderate positive correlation (*r*=0.46 and *r*=0.34, respectively) with improvement in daily fruit and vegetable intake. Among social support for healthy diet subscales, increased friend encouragement for a healthy diet had a moderate positive correlation (*r*=0.43) with an increase in daily fruit and vegetable intake. A moderate negative correlation (*r*=−0.25) was seen between perceived barriers to healthy diet and daily fruit and vegetable intake (ie, greater barriers to healthy diet corresponded with less fruit and vegetable intake). For PA, 2 subscales of social support for PA (family and friend participation) had a moderate positive correlation (*r*=0.51 and *r*=0.61, respectively) with MPA per week.

**Table 3:** Correlations of changes in diet-related psychosocial measures to changes in cardiovascular health behaviors (N=45).

Diet-related psychosocial measures			Correlation to change in daily fruit and vegetable intake^a^ (servings per day)
Diet self-regulation			
	Increase fruit, vegetable, and grain intake	0.46	
	Decrease fat and calorie intake	0.03	
	Nutrition tracking	0.34	
Social support for healthy diet			
	Family encouragement	0.004	
	Family discouragement	0.19	
	Friend encouragement	0.43	
	Friend discouragement	0.05	
Perceived barriers to healthy diet			−0.25

^a^Correlations >0.2 were considered moderately associated.

**Table 4:** Correlations of changes in physical activity (PA)-related psychosocial measures to changes in cardiovascular health behaviors (N=45).

PA-related psychosocial measures		Correlation to change in MPA^a^ per week^b^ (minutes per week)
PA self-regulation		0.17
Social support for PA		
	Family rewards or punishment	0.09
	Family participation	0.51
	Friend participation	0.61
Perceived barriers to PA		−0.08

^a^MPA: moderate-intensity physical activity.

^b^Correlations >0.2 were considered moderately associated.

### Comparisons With Covariates

In multivariable regression analyses, there were no statistically significant differences in pre- and postintervention score changes among any of the sociodemographics, EHL, or app engagement groups for diet and PA-related psychosocial measures and CVH behaviors.

## Discussion

### Principal Findings

Our findings demonstrate that a culturally tailored, community-based mHealth lifestyle intervention can improve key diet and PA-related psychosocial factors and CVH behaviors in African Americans. In addition, several of the improvements in these diet and PA-related psychosocial measures were associated with improvements in diet and PA, which to our knowledge has not been previously described in the setting of a mobile app–based lifestyle intervention. The changes noted in our study were of small to medium effect sizes. This is consistent with the findings of other studies with similar sample sizes evaluating changes in diet and PA-related psychosocial measures [40]. A longer intervention timeframe may be needed to see greater changes in the diet and PA-related psychosocial measures evaluated in this study.

Our findings illustrating the benefits of using an mHealth lifestyle intervention on diet and PA-related self-regulation in African Americans are consistent with those of other investigators. Participants in our study reported improvements in the subscales of diet self-regulation as well as PA self-regulation. Furthermore, improvements in diet self-regulation subscales were positively associated with increased daily fruit and vegetable intake. Ferrante et al [34] evaluated changes in diet and PA-related psychosocial variables and weight loss outcomes in a Fitbit plus mobile app lifestyle intervention versus a Fitbit only control group among African American survivors of breast cancer. Compared with the control group, participants in the intervention arm demonstrated sustained improvement across a larger number of self-regulatory behaviors for healthy diet and PA. In the *Smart Walk* study, an mHealth app–based intervention aimed at increasing PA in African American women, participants demonstrated improvements in self-regulation for PA [40]. Similar to these cohorts, our analytic sample was predominantly composed of African American women. Qualitative data on the interplay between health and spirituality in African American women show that health self-management in African American women is deeply intertwined with their spiritual and faith connections [63]. Thus, it is possible that faith-based interventions, such as this study, may provide greater self-regulatory benefits for African American women.

Social support has been consistently identified as an important psychosocial variable for maintaining healthy diet [64] and PA, with family members and friends being identified as key sources of support [14,17]. Among the social support subscales for healthy diet, participants reported a decrease in friend discouragement in our study. In addition, improvements in friend encouragement were associated with increased daily fruit and vegetable intake. With respect to social support for PA, improving trends were noted for friend participation; however, this did not reach statistical significance. However, increases in family and friend participation were positively associated with increased MPA per week. Ferrante et al [34] reported improvements in social support for healthy diet but no significant changes in social support for PA, similar to our findings. In the *Smart Walk* study, there was no significant improvement in social support for PA; however, qualitative participant feedback suggested that increasing features for engagement on a sharing board with moderated discussion by the study team can facilitate participation and possibly increase social support [40]. In a recent qualitative analysis of the FAITH! app pilot study, participants demonstrated that they received encouragement and social support toward a healthy lifestyle from posts by other participants on the sharing board [48]. Thus, facilitating opportunities for discussion among study participants through a sharing board feature may foster meaningful engagement in mHealth interventions and increase social support in mHealth lifestyle interventions. Further investigation is necessary to better elucidate the most effective means of enhancing social support through greater opportunities for engagement within mHealth interventions.

Prior studies have pointed to multiple barriers such as fatigue, time, cost, and lack of social support leading to poor diet and PA among African Americans [13,14,16]. In addition, there are links between socioeconomic and educational inequities experienced by African Americans that influence perceived barriers to healthy diet and PA. Analysis of survey data from a predominantly African American population by Sharpe et al [65] found that food-secure households reported better diet-related psychosocial factors than food insecure households; however, both groups had largely similar dietary intake patterns. Another study by Wilcox et al [66] found that in a predominantly African American population, less than a high school education was associated with lower diet quality, whereas income and food security were positively associated with higher diet quality. Employed African Americans have been found to have a lower cooking frequency than unemployed African Americans, which indicates that time constraints may be a limiting factor in healthy diet among employed African Americans [16]. Our evaluation of participants’ perceived barriers to healthy diet and PA encompassed similar factors as these studies but also included sociocultural considerations (hairstyle), ability to prepare healthy meals, environmental constraints (neighborhood), and support from family and friends. Overall, there was an improvement in perceived barriers to healthy diet, which further correlated with improvements in dietary intake. These findings suggest that our mHealth lifestyle intervention may offer support to African Americans in the navigation of perceived barriers stemming from longstanding structural inequities—or possibly, in spite of these existing inequities—by providing CVH education and highlighting practical strategies to incorporate healthy diet into daily life.

### Strengths and Limitations

There are several strengths to this study. Our findings contribute further data on improvements in diet and PA-related psychosocial factors in African Americans participating in an mHealth lifestyle intervention. We further provide novel contributions by describing how changes in these underlying diet and PA-related psychosocial factors are associated with diet and PA behaviors. The intervention was co-designed with African American community members from participating churches who could give voice to the daily lived experiences of African Americans to ensure that the intervention emphasized African American faith, spirituality, and social connectedness. This study implemented an mHealth lifestyle intervention that was well-aligned with smartphone use patterns in African Americans and one that was well-received by participants [48].

Our study has several limitations. This pilot study included a small convenience sample of African Americans residing in Minnesota; thus, our study was limited by selection bias and is not representative of all African Americans. Furthermore, the small sample size limited our statistical power. As such, we were unable to run formal mediation analyses to probe causal relationships between diet and PA-related psychosocial factors and their respective behaviors. With respect to covariate comparisons, our sample was not adequately sized to detect meaningful patterns based on sociodemographics, EHL, or level of app engagement. The psychosocial factors and CVH behavior measures were self-reported by the participants, which could reflect social desirability bias. Our pretest-posttest, quasi-experimental design lacked a control group, and the study was of a relatively short duration. A longer intervention duration may demonstrate a greater effect on diet and PA-related psychosocial measures. Research is currently underway to further evaluate the preliminary findings presented in this pilot study with a larger, more representative sample of African Americans within a randomized controlled trial (NCT03777709).

### Conclusions

Our preliminary findings indicate that the use of a culturally tailored mHealth lifestyle intervention can improve diet and PA behaviors as well as several underlying diet and PA-related psychosocial factors. Diet and PA-related self-regulation, social support, and perceived barriers may be key psychosocial variables to target in future mHealth lifestyle interventions aiming to improve CVH behaviors among African Americans. In addition, we co-designed and implemented a mobile app–based intervention in partnership with an underserved African American community that was well-aligned with and complemented their mobile technology use patterns. It is important to understand the nuances of mobile technology use among African Americans compared with other populations in the United States and to design interventions that account for these differences to prevent the widening of the digital divide. Mobile app–based interventions may be powerful tools to address CVH disparities that disproportionately affect African Americans.

## Abbreviations

CVD: cardiovascular disease
CVH: cardiovascular health
EHL: eHealth literacy
FAITH!: Fostering African American Improvement in Total Health
LS7: Life’s Simple 7
mHealth: mobile health
MPA: moderate-intensity physical activity
PA: physical activity
